# Applying the Strategic Health Purchasing Progress Tracking Framework: Lessons from Nine African Countries

**DOI:** 10.1080/23288604.2022.2051796

**Published:** 2022-03-01

**Authors:** Agnes Gatome-Munyua, Isidore Sieleunou, Edwine Barasa, Freddie Ssengooba, Kaboré Issa, Sabine Musange, Otieno Osoro, Suzan Makawia, Christelle Boyi-Hounsou, Eugenia Amporfu, Uchenna Ezenwaka

**Affiliations:** a Department of Health Portfolio Results for Development, P.O.Box 389 - 00621 Nairobi, Kenya; b Department of Health Research, Research for Development International, Yaounde, Cameroon; c Health Economics Research Unit, KEMRI Wellcome Trust Research Programme, Nairobi, Kenya; d Department of Health Policy Planning & Management, Makerere University School of Public Health, Kampala, Uganda; e Department of Health Research, Recherche pour la Santé et le Développement (RESADE), Ougadougou, Burkina Faso; f School of Public Health, University of Rwanda, Kigali, Rwanda; g Department of Economics, University of Dar es Salaam, Dar es Salaam, Tanzania; h Department of Health System, Policy and Economic Evaluations Ifakara Health Institute, Dar es Salaam, Tanzania; i Department of Health Research Centre de Recherche en Reproduction Humaine et en Démographie (CERRHUD), Cotonou, Benin; j Department of Economics, Kwame Nkrumah University of Science and Technology, Kumasi, Ghana; k Health Policy and Research Group, University of Nigeria, Enugu, Nigeria

**Keywords:** Africa, health purchasing, purchasing framework, strategic health purchasing, strategic Health Purchasing, Progress Tracking, Framework

## Abstract

The Strategic Purchasing Africa Resource Center (SPARC) developed a framework for tracking strategic purchasing that uses a functional and practical approach to describe, assess, and strengthen purchasing to facilitate policy dialogue within countries. This framework was applied in nine African countries to assess their progress on strategic purchasing. This paper summarizes overarching lessons from the experiences of the nine countries. In each country, researchers populated a Microsoft Excel–based matrix using data collected through document reviews and key informant interviews conducted between September 2019 and March 2021. The matrix documented governance arrangements; core purchasing functions (benefits specification, contracting arrangements, provider payment, and performance monitoring); external factors affecting purchasing; and results attributable to the implementation of these purchasing functions. SPARC and its partners synthesized information from the country assessments to draw lessons applicable to strategic purchasing in Africa. All nine countries have fragmented health financing systems, each with distinct purchasing arrangements. Countries have made some progress in specifying a benefit package that addresses the health needs of the most vulnerable groups and entering into selective contracts with mostly private providers that specify expectations and priorities. Progress on provider payment and performance monitoring has been limited. Overall, progress on strategic purchasing has been limited in most of the countries and has not led to large-scale health system improvements because of the persistence of out-of-pocket payments as the main source of health financing and the high degree of fragmentation, which limits purchasing power to allocate resources and incentivize providers to improve productivity and quality of care.

## Introduction

Health purchasing is the allocation of pooled funds to health providers for the delivery of health services on behalf of the population and is one of the functions of health financing systems.^1,2^ Purchasing is increasingly recognized in public discourse and literature as an important lever that health financing systems can use to make the most effective use of limited health resources.^3–5^ Purchasing can affect the achievement of health system objectives and progress toward universal health coverage (UHC)^1^ by improving equity and efficiency in resource distribution and use as well as quality of health care services.^1,4,6,7^

Purchasing can be passive or strategic. Passive purchasing transfers pooled funds based on historical expenditure or norms (such as number of beds or health workers rather than what services are needed by populations and how much funding is needed to deliver those services) or, on the other extreme, it involves open-ended fee-for-service payments with no mechanism for expenditure management.^3,8^ Passive purchasers transfer funds without considering the selection of the most efficient and effective providers and do minimal monitoring of quality standards.^4,8^

Strategic purchasing, in contrast, means deliberately directing health funds to priority populations, interventions, and services. It involves actively creating incentives so funding is used equitably and efficiently and is aligned with population health needs; using information to make decisions about what health services should have priority for public funding; selecting providers from whom these health services can be accessed; and defining how and how much will be paid to providers to deliver those services.^3–6^

A strong strategic health purchasing system has a set of core functions—benefits specification, contracting arrangements, provider payment, and performance monitoring—that are supported by clear governance arrangements that allocate responsibility for carrying out the purchasing functions.^9–11^ Strategic purchasers aim to define the services and service guidelines and set the price and quality of services they are willing to pay for on behalf of the covered population. This is defined in contracts that have clear service delivery guidelines, quality and performance measurement benchmarks, and performance monitoring of providers to ensure that they meet the contractual obligations.

**Figure 1:**
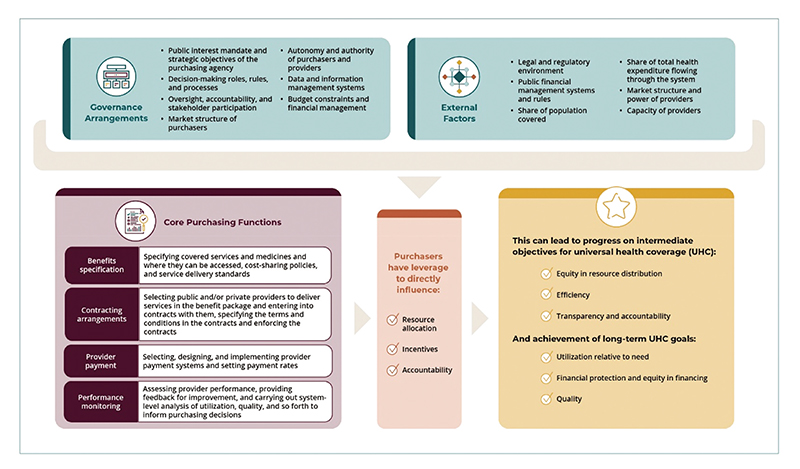
Strategic Health Purchasing Progress Tracking Framework.

The Strategic Purchasing Africa Resource Center (SPARC), a resource hub and a consortium of technical partners, co-created a practical framework for describing, assessing, and improving purchasing: the Strategic Health Purchasing Progress Tracking Framework (Figure 1).^12^ The framework is accompanied by a Microsoft Excel spreadsheet to guide data collection to map health purchasing systems at the country level, as well as benchmarks drawn from the published literature and normative guidance in existing frameworks, to assess how strategically the functions are being carried out. The framework was applied in nine African countries—Benin, Burkina Faso, Cameroon, Ghana, Kenya, Nigeria, Rwanda, Tanzania, and Uganda—to describe purchasing across health financing arrangements, identify progress toward strategic purchasing, and assess the impact of purchasing on UHC objectives. The overall objectives were to inform country-level policy priorities and actions and contribute to the regional strategic purchasing learning agenda. This paper describes the application of the framework in the nine countries, areas of progress in strategic purchasing, and some of the obstacles countries need to overcome so they can continue to improve strategic purchasing to advance UHC goals.

## Methods

The Strategic Health Purchasing Progress Tracking Framework was used to make a descriptive cross-sectional study of the purchasing functions across all main health financing arrangements in each of the nine countries. Technical partners based in those countries—10 across the nine countries—undertook the study. They selected four to six of the most significant health financing schemes in their country and mapped the purchasing functions according to the framework. Criteria for identifying the health financing schemes to examine—those with the most leverage to advance progress toward UHC—included total health expenditure flowing through the scheme, population covered by the scheme, and whether a scheme had been identified in policy documents as being a main vehicle for achieving UHC. The functional mapping exercise involved delving into the details of the mandates and roles of different institutions and stakeholders for each purchasing function and how they interact within and across health financing arrangements.

### Data Collection

The technical partners collected information to populate the Excel data collection tool between September 2019 and March 2021. The tool guides collection of descriptive information about governance arrangements (such as legislative instruments, declarations, and regulations that define the mandates of purchasers and providers), how the purchasing functions are carried out (including the institutional roles, policies, and specific processes implemented by each institution), external factors that affect purchasing (such as legislative instruments and regulations and rules outside the purview of the health sector that affect purchasing functions), and verifiable data on the intermediate results achieved through the purchasing functions and any demonstrable effects on UHC intermediate objective and long-term goals. The technical partners began with an extensive document review of gray and published literature, including government strategy documents, government reports, declarations, relevant legislation, program reports, and audited accounts of health insurance schemes and ministries of finance. Gaps in the literature were supplemented with key informant interviews to validate what was captured in the document review. All participants were informed of the objectives of the study, why they were selected for interviews, and that their participation was voluntary. Each informant provided written informed consent.

### Data Analysis

The technical partners analyzed the descriptive data and identified emerging themes in their country.^13,14^ SPARC used an in-person workshop to bring together all of the partners and validate these themes, identify common themes across countries, and describe the commonalities further to draw out similarities and differences in country experiences. These themes were further validated with the technical partners and policy makers from each country to ensure their accuracy.

### Study Setting

The nine countries reflect the regional diversity of the continent and include four in East Africa, one in Central Africa, and four in West Africa. Five are Anglophone countries, two are Francophone, and two bilingual. They represent a mix of income categories, with three low-income and six lower-middle-income countries. Table 1 presents the nine countries’ health financing indicators. Health spending varies, with current health expenditure between $37 USD and $88 USD per capita and generally low public spending on health. Service coverage is low, with seven of the nine countries below 50% coverage. Out-of-pocket spending varies widely, between 11% and 77%, and although the incidence of catastrophic spending may be low, this may be due to households forgoing care and may not capture unmet need due to direct and indirect costs of accessing health care.

## Results

A common finding across the nine countries is a high degree of fragmentation across health financing arrangements, with multiple schemes and purchasers—including government budgets, national health insurance, voluntary private and community-based health insurance, occupational health insurance, and donor-funded schemes. For example, 30 schemes were identified in Cameroon, while Kenya had more than 70 schemes under the National Hospital Insurance Fund (NHIF).^13,14^ Each health financing scheme has its own purchasing functions, with different agencies involved.

The existence of multiple schemes results in fragmented funding flows; multiple benefit packages; duplication of coverage for some population groups and gaps for other groups; multiple payment methods and financing flows to providers, often with conflicting incentives; and multiple fragmented information systems. Donor-funded projects, such as vertical programs and performance-based financing (PBF) schemes, are often implemented in parallel rather than integrated to existing schemes.^15^ The impact of fragmentation and multiple financing flows has been studied in Kenya and Nigeria.^16–19^ Multiple funding flows provide incentives to health providers to shift costs, leading to a two-tier health system in which clients in some schemes are preferred by providers to others.

Devolved systems of government lacking effective governance structures that articulate the roles of each level of government, and foster coordination toward national objectives, tend to worsen this fragmentation. In decentralized settings, the power of national purchasers may be diluted because subnational governments have authority over many decisions that affect resource allocation and incentives at the local level. This is the case in Kenya and Nigeria, where fragmentation across financing arrangements at the national level is compounded by fragmentation across counties (Kenya) and states (Nigeria), resulting in high administration costs and further reducing the amount of funds that reach providers to provide health services to beneficiaries.^20^

### Progress in Strategic Purchasing

**Figure 2:**
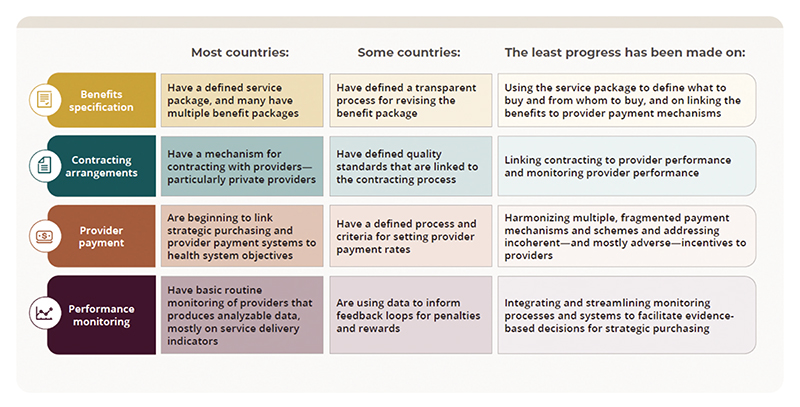
Summary of country level-findings by purchasing function.

In terms of progress within specific purchasing functions, all nine countries have made some progress in specifying a benefit package that addresses the health needs of the most vulnerable groups, and although most of the countries have multiple benefit packages, some are taking steps to harmonize the packages into a single UHC benefit package. Informal arrangements predominate for contracting with public providers, but selective contracting based on provider capacity and quality benchmarks, occurs mostly with private providers to clarify expectations and priorities. Progress on provider payment and performance monitoring has been limited. Figure 2 summarizes the country-level findings by purchasing function.

### Benefits Specification: Improve Access to Priority Services

Benefit package specification has been used as a tool to define priorities, reflect health system goals of equitable access, and protect entitlements for priority populations.^21^ The *Gratuité* program in Burkina Faso and Nigeria’s Basic Health Care Provision Fund (BHCPF) are publicly funded programs that focus on low-cost, high-value services such as reproductive, maternal, neonatal, and child health (RMNCH) services, including family planning. These programs target to improve maternal and child health indicators by addressing priority RMNCH services. Inclusion of these services in the benefit package has increased resources allocated to these services and has improved access to RMNCH services by reducing financial barriers.^22^

For example, a precursor to the BHCPF in Nigeria known as the Subsidy Reinvestment and Empowerment Program (SURE-P) Maternal and Child Health Initiative, a national program that ran for four years from 2012 to 2015, provided a benefit package that included high-priority RMNCH services.^23^ This focus led to a 36.3% increase in number of pregnant women attending four or more prenatal visits and a 32.1% increase in the number of pregnant women delivering with a skilled birth attendant present.^23^

In Benin, *Assurance pour le Renforcement du Capital Humain* (ARCH)—the flagship social protection scheme that provides health insurance primarily to the poor—developed a benefit package based on three criteria: 1) priority public health challenges, including malaria and tuberculosis; 2) the cost of services; and 3) an impact assessment of different resourcing models. Based on these criteria, the benefit package targets the management of common conditions, childbirth, and treatment of children under age 5.

Tanzania’s National Health Insurance Fund and Kenya’s National Hospital Insurance Fund use benefits specification and contracting, to clearly specify entitlements included in RMNCH services. Contracts include requirements to adhere to service guidelines and protocols, which are then used for assessing claims prior to payment and for performance monitoring of providers through medical audit of claims. The Linda Mama Program in Kenya aims to increase access to skilled healthcare providers for child delivery and provides access to good quality services through a wide range of public and private providers through selective contracting.^24^

Selecting what is covered and not covered in the benefit package requires tradeoffs. Explicit priority-setting processes are necessary to create transparency and accountability in these decisions. In Kenya, a health benefits advisory panel established by the Ministry of Health (MOH) has a well-defined process for identifying explicit criteria for the selection of services.^25,26^ The panel includes the MOH, public and private purchasers, public and private providers, civil society organizations, and patient groups.^26^ Similarly, in Benin, the benefits specification process involved a discussion between MOH and politicians to reach consensus on the benefit package for the ARCH scheme. Community engagement in the design of benefit packages for community health *mutuelles* in Benin and occupational health *mutuelles* in Burkina Faso has been common practice. Annual general meetings and follow-on customer and patient feedback surveys have also been used to engage beneficiaries in discussions about benefits that should be included and providers that should be selected. Ghana, Rwanda, and Nigeria are embarking on priority-setting processes and setting up health technology assessment systems to institutionalize benefits specification going forward.

### Contracting Arrangements: Create a Culture of Accountability

Contracting arrangements, including performance agreements and intergovernmental agreements, define institutional roles and responsibilities, balance power across stakeholders, and define mechanisms to hold all sides accountable for delivering on their responsibilities. Contracting is most useful for strengthening accountability between purchasers and providers when contracts clearly specify the benefits to be provided, payment rates, service delivery requirements, referral and gatekeeping guidelines, and mechanisms for redress. Provider contracts can be a way to influence provider behavior and incentivize the provision of quality health services. To do this, they should be specific and linked to clear health system objectives and accountability measures.

In health care, individuals seeking services may not have perfect information to make the best decisions about where to access care, which interventions are most appropriate, and how much those interventions should cost.^27–29^ This leads to information asymmetries and “principal-agent relationships” in which the principal recognizes the need for an agent to act on their behalf and make decisions that maximize their welfare.^7,30^ Four key actors are involved in these principal-agent relationships: individuals, communities, or households; purchasers; health care providers, and the government or regulators.^31,32^ Creating a culture of accountability through contracting arrangements can improve principal-agent relationships between the purchaser and citizens, the purchaser and providers, and purchasers and regulators.^33,34^ Rwanda’s annual contracting process, involving the MOH and district administration, health facilities, and health workers, stipulates service delivery targets; this creates a culture of accountability for health system results that cascades upward from the district level to the national level.^35^ The MOH and districts verify achieved targets at the end of the contracting cycle.

In Ghana, an autonomous agency called the Health Facility Regulatory Agency (HeFRA) registers, inspects, and licenses all health facilities. HeFRA registration is a prerequisite for contracting by the NHIA that manages the NHIS. The NHIA contracts providers through provider associations for example, Ghana Health Services (GHS) for public health facilities and Christian Health Association of Ghana (CHAG) for faith-based health facilities. Individual providers to be included under the contract are credentialed by the NHIA and a list of credentialed providers is included as an appendix to the contract. This reduces transaction costs as one contract is signed on behalf of all providers under these umbrella bodies.^36^ Contracts include the services and medicines covered by the scheme, tariffs, claims submission, quality standards, time frame of the contract (usually one year), and termination clauses. To improve accountability and quality of care, NHIA has introduced quality benchmarks in provider contracts, such as average length of stay and minimum readmission period for in-patient services. As an example, readmission within three days of the last admission, is an indicator of poor quality of care and/or early discharge and the hospital claim is rejected.

In Cameroon, Rwanda and Uganda, provider contracts for the PBF programs go beyond listing expected services and payment terms, to include requirements for data collection and provider performance monitoring with specific targets (linked to the national targets for maternal child health services) that should be met by providers, and sanctions/penalties for noncompliance. These additional requirements have resulted in better adherence by providers and have improved access to quality health care services. Although these PBF programs are usually donor funded, they have introduced the contracting function at both the purchaser and provider levels and have created a foundation and local capacity for strategic purchasing that can be built on in future national schemes.^37^

Many government-budget-financed schemes use less formal contracting between the Ministry of Finance (MOF) or MOH and providers. In Kenya and Uganda, intergovernmental agreements are used between the national government and devolved government units that are responsible for service delivery, and they define roles and responsibilities between levels of government.^38,39^ In Uganda, the MOH uses soft tools such as memorandums of understanding (MOUs) rather than explicit contracts with private nonprofit providers. Although these MOUs are less explicit, less formal, and have limited enforceability, they create a culture of contracting and initiate a process of building trust.^40^ The private sector is increasingly recognized as an important channel through which to expand access to services. For example, contracting with private providers in Burkina Faso, particularly in urban areas where they are commonly located, has expanded access to services.^41^ Contracting has been used as a tool to specify the cost of services and ensure that payments are kept at a fair and sustainable level.^41^

In several countries, citizen and civil society representation can help ensure that purchasing arrangements and processes—including benefits specification, selection of providers, and performance monitoring—account for the preferences of beneficiaries and hold accountable the agents acting on their behalf. In Burkina Faso, international nongovernmental organizations (INGOs) play a key role in accountability for the publicly funded *Gratuité* program. Providers are paid in advance for services and submit claims to the district.^41^ The INGOs review a sample of these claims and report any discrepancies between services delivered and amount paid. The MOH uses this report to reconcile payment in the next funding cycle by either increasing or decreasing disbursements, as the case may require. In Tanzania, community representation on health facility governance committees ensures a role for citizens in facility planning and budgeting, oversight of budget execution and accounting processes. Through these committees, citizens hold facility managers and staff accountability for providing quality services. In Uganda, health unit management committees (HUMCs), which comprise community representatives, play a similar role, but their effectiveness is limited by poor selection of representatives and lack of training.^42,43^

Weak accountability, on the other hand, can undermine the results of strategic purchasing. When Benin set up ARCH, the government created a purchaser-provider split to separate functions and clearly delineate the roles and mandates of the purchaser and providers. However, a power imbalance has remained, with providers having a limited voice in establishing contracts, setting payment rates, and agreeing on performance monitoring indicators. This puts providers at a disadvantage, such as when claims payments are delayed, and no effective mechanisms exist to hold the purchaser accountable for meeting the terms of the contract.

Weak accountability of the purchaser to meet contractual obligations to providers in a timely manner also reduces the impact of strategic purchasing in Kenya and Ghana. In Kenya, delays in provider payment reduce access to services by leading providers to levy informal charges and underprovide services.^18^ In Ghana, delays in provider payment have been a key factor in providers charging informal fees to NHIS beneficiaries.^44^

### Provider Payment: Improve Resource Allocation and Get Funds to Frontline Providers

Government budget financing—channeled from the MOF to the MOH, the purchaser—is the predominant source of funds for purchasing health services for the majority of the population in sub-Saharan Africa. Longstanding national health insurance schemes, such as in Kenya and Tanzania, account for only a small share of total health expenditure and cover a small share of the population, mostly wealthier people.^45^ This makes purchasing through the government budget particularly important for achieving equity goals. Even in Ghana and Rwanda, where the NHIS has reached significant population coverage, less than 15% of total health expenditure flows through the NHIA and Rwanda Social Security Board (RSSB), respectively, and the majority of funding comes from government budgets.^46,47^ A limitation of government budgets is the high proportion committed to recurrent expenditure, including wages, which are often dictated outside of the health sector by the civil service system. There is often little remaining that is discretionary and can be allocated to medicines, supplies, and other inputs to improve quality of care.^48^ Further, the ratio of recurrent budgets to capital expenditure is 8:1, leaving few resources for the development of health infrastructure.^49^

Government budgets channeled through input-based, line-item budgets continue to be a significant means of allocating resources and paying health care providers in many African countries, so they need to be used as a tool to improve purchasing. Although input-based budgets have constraints, a good budget structure with public financial management (PFM) rules that allow autonomy and flexibility can facilitate strategic purchasing of limited resources and also create a system of accountability for achieving health system goals.

Tanzania has harmonized multiple sources of funding from government budget financing (with on-budget donor support) into the Health Sector Basket Funding (HSBF).^50^ The Direct Health Facility Financing (DHFF) introduced in 2018, applies a single allocation criterion using capitation payment to transfer HSBF to providers. This provides a coherent set of incentives to providers and reduces their accounting burden. Tanzania is overcoming rigid PFM rules by recognizing frontline primary health care (PHC) providers as independent accounting units and transferring flexible funds through DHFF that providers can use to respond to community-level needs. Community participation in budget development on health facility governing committees strengthens the link between community preferences and the budgeting process, while referencing the budget guidelines provided. Tanzania has developed clear budget guidelines and accountability mechanisms for funds transferred to PHC providers. Through these funds, facilities have been able to make minor renovations to facility infrastructure, purchase medical commodities, and pay for operational expenses such as travel, staff overtime, and other incentives.

Uganda sends funds directly to lower-level health facilities, which has led to more timely provision of funds to those facilities. But the funds are conditional, with strict criteria based on line items on how these funds can be used, which limits the ability of facilities to use the funds to meet local needs. In addition, the amount sent varies from $558 USD at the Health Center II level to $2,235 USD at the Health Center IV level in financial year 2020/2021, and it is much lower than facilities need, limiting the effectiveness of direct funding to facilities.^51,52^

Burkina Faso’s *Gratuité* program overcame PFM rigidities by allowing health facilities to set up bank accounts to receive government budget funds through the program. The MOH allows for advance payment to health providers based on their previous quarter’s claims. Reconciliation of payments is verified by INGOs, which are funded through the government budget, limiting fraud and misappropriation of funds. An e-*Gratuité* platform is used for financial and service reporting and provides information for performance monitoring and making purchasing decisions.

In Uganda, program-based budgeting has improved the use of government funds by linking the budget to predetermined national priorities and district needs. Compliance certificates from the National Planning Authority ensure that budget centers, including local governments, align with national priorities. Uganda’s Public Finance Management Act of 2015 details procedures and sanctions to reinforce accountability, while direct funds transfers to PHC facilities via PHC grants offset bureaucratic delays and leakages at the district level.^53^ Supportive performance monitoring by districts ensures that corrective action is taken.

In Uganda and Kenya, program-based budgeting helps link the budget to health system objectives and allocate funds across programs to priority services. Adoption of program-based budgeting and planning has allowed evolution from paying providers for inputs to purchasing outputs and outcomes. Program-based budgeting has loosened some of the rigidities of line-item budgets in these countries by providing flexibility in the spending of funds within the larger program. Although program-based budgeting has encountered implementation challenges, it has been a key mechanism for overcoming some PFM rigidities in how funds are budgeted and spent.

### Performance Monitoring: Improve the Collection and Use of Analyzable Data

Most countries have rudimentary health management information systems, and often multiple systems that are not interoperable or integrated, which results in duplication, fragmentation and limited use for decision making. There is increasing recognition that these systems need improvement and better linkages to purchasers, so purchasers can make better-informed purchasing decisions. This has happened in a few cases. Burkina Faso’s *Gratuité* scheme uses the e-*Gratuité* platform for provider monitoring, which is hosted on District Health Information System 2. Providers submit their activity reports and claims through e-*Gratuité*. INGOs verify claims on the platform against paper-based claims. MOH reconciles claims after the review, and correct overcharges and undercharges in the next disbursement. The MOH stops payment to providers that do not submit monitoring reports for three consecutive months.

In Tanzania, the introduction of DHFF has gone hand in hand with improved provider monitoring, through the Facility Financial Accounting and Reporting System (FFARS), which links budgets to their execution. FFARS provides a systemwide view from the facility level to districts, regions, and the national level and provides valuable data for purchasing decisions on budget allocation and budget tracking.^54^

PBF has had mixed results in improving the availability and use of data for purchasing decisions. The donor-driven nature of many PBF programs has resulted in a proliferation of contracting arrangements, provider payment methods, and verification systems that are external to the government budget system.^55^ Although studies from Cameroon, Kenya, Rwanda, and Uganda have shown improvements in access to RMNCH services, there have been unintended consequences including the crowding out of other services that are not incentivized in the PBF program.^37^ Although PBF has set indicators and targets that are used for performance monitoring, care must be taken so stakeholders do not pick only the indicators that benefit them or provide financial incentives to them and exclude indicators that provide information for systemwide improvement.

Information and communications technology (ICT) is being used to improve operational efficiencies. Ghana has invested in a number of ICT improvements to the NHIS, including automating claims processing, provider credentialing, receipt of funds to NHIA, and mobile phone renewals. Efficiency gains are expected from reducing human intervention in these processes and reducing the burden of handling and storing paper-based claims, thereby reducing administration costs. The convenience and ease of mobile phone renewals may increase the renewal ratio and premiums received through the mobile channel.

### Governance: Reduce Fragmentation to Increase Purchasing Power

The countries that have made the most progress in improving strategic purchasing have been able to reduce fragmentation in their systems, thereby increasing the power of the main purchasing agency to influence resource allocation, incentives, and accountability in the system. Ghana and Rwanda have reduced fragmentation within former community-based insurance schemes by merging multiple schemes at the district level into a national system with consolidated purchasing power, which has advanced progress on strategic purchasing for UHC.^56,57^ Both countries have a dominant purchaser—the NHIA in Ghana and RSSB in Rwanda. In Rwanda, the consolidation of the schemes involved a two-phase process, starting with the merging of the schemes under MOH management and the transfer of the scheme to RSSB, which also manages the RSSB scheme for formal-sector workers.^57^ The NHIA and RSSB have well-defined mandates for the purchasing functions of selective contracting and provider payment.

In Ghana, the NHIS, managed by NHIA, now covers 40% of the population and accounts for 11.4% of total health spending in the country; in Rwanda, 85% of the population is covered by the schemes under RSSB, with 13% of total health spending flowing through the schemes.^46,47,58^ Although they still manage only a small share of total health spending in their countries, these agencies have been able to use their purchasing power—through selective contracting, institutionalized gatekeeping, and performance monitoring systems—for evidence-based decision making.^36,59^ The purchasing agencies in Ghana and Rwanda have not made full use of their purchasing power to introduce close-ended payment systems as they pay providers through open-ended diagnosis-related groups in Ghana and fee-for-service in Rwanda, without budget caps. As a result, the schemes in both countries face financial sustainability challenges.^36,59^

Other countries are taking creative steps to reduce fragmentation by pooling resources from government budget financing with donors’ on-budget resources, as in the case of Tanzania’s HSBF.^50^ Rwanda’s PBF program consolidates PBF funds from different donors with government resources to provide a more harmonized approach to incentivizing providers.^59^ In Uganda, fragmentation and duplication of purchasing functions are reduced by assigning donors to specific regions to avoid duplication and overlap of services.^60^

## Discussion

This study identified pockets of progress on strategic purchasing in all nine countries, through improved benefits specification, contracting arrangements, provider payment, and performance monitoring. However, this progress has largely been within individual schemes and has not led to large-scale health system improvements in most cases—because of the low level of government health spending overall, the persistence of a high share of out-of-pocket spending, and a high degree of fragmentation across health financing schemes.^13,14^ High out-of-pocket expenditure and inadequate pooling of funds reduces purchasing power to exert influence over resource allocation, incentives to providers, and accountability throughout the system. The high level of fragmentation, with multiple uncoordinated purchasers, often leads to duplicative purchasing functions and incoherent incentives to providers. Some of the fragmentation is due to multiple donor-funded projects with duplicative purchasing functions.

All of the countries in the study need to address the challenges of low government health spending, lack of pooling, and fragmentation, if they are to use purchasing to advance progress toward UHC. These are largely political challenges, and political solutions are required to overcome them. They may be beyond the typical scope of strategic purchasing technocrats. Good stewardship and political will, broad-based stakeholder consultation and engagement, and a clear multi-year strategy will be needed to overcome these obstacles.^61^

The countries also face technical challenges with purchasing policy—such as introducing effective provider payment systems and using performance monitoring to inform purchasing decisions—and operational challenges such as paying health care providers on time and reducing administrative burden in claims processes. Although most of the countries have clear mandates for purchasers enshrined in policy documents and legislative instruments, often the implementation of the governance arrangements has been weak or conflicting laws have resulted in duplicative or overlapping functions, resulting in conflicts.^60^

Four key lessons from this study may be useful for other countries in seeking to overcome obstacles and plot a course toward more higher-impact strategic purchasing:

(1) Defragment and consolidate purchasing functions across schemes
(2) Make incremental improvements to government budget financing
(3) Allocate funds to frontline providers and increase their autonomy to respond to incentives
(4) Build on existing information systems to generate evidence for purchasing decisions

### Defragment and Consolidate Purchasing Functions across Schemes

Fragmentation not only inhibits strategic purchasing, but it reduces the effect of cross-subsidization from the rich to the poor and the healthy to the sick, leads to variable entitlements, and entrenches inequalities in access.^62–64^ Ghana and Rwanda provide lessons on effective consolidation of smaller schemes into a larger pooling mechanism that enhances equity and consolidates purchasing power. Although the full extent of purchasing power has not been tapped by the RSSB in Rwanda, the country has achieved impressive coverage and reduction in out-of-pocket spending to only 11% of total health expenditure.

Countries do not have to wait until major consolidation of health financing arrangements becomes politically feasible to take steps toward defragmenting purchasing arrangements. Strengthening alignment of purchasing functions across the different health financing schemes and harmonizing the various purchasers and health spending functions (such as the budget for salaries, procurement of medicines, and operational and maintenance budgets) can reduce fragmentation and improve the efficiency of resource use even without top-down consolidation or integration of schemes. In Tanzania, different funding streams, including donor funds and government budget funds, are consolidated at the level of the health care provider through DHFF. In Rwanda, consolidation efforts are starting for accreditation of providers for contracting in the community-based health insurance scheme managed by RSSB and in the PBF scheme managed by the MOH.

Further, PBF has been conflated with strategic purchasing in many low- and low-middle income countries,^65^ and conceptual clarity is needed to distinguish between PBF and strategic purchasing. PBF is not the same as strategic purchasing, but PBF can be used as a tool to improve purchasing functions—by clearly stipulating benefits, linking benefits and quality assurance in contracting arrangements, linking provider payment to health system objectives, and improving performance monitoring to track provider behavior and align incentives.^37,55,66^ Rather than worsening fragmentation, PBF can be aligned with government funding streams to create cohesive incentives to providers to link outputs to health system objectives.^55^

### Make Incremental Improvements to Government Budget Financing

Many countries have aspirations for setting up new health financing arrangements—usually national health insurance schemes, which are gaining prominence in sub-Saharan Africa.^67^ But these schemes often take significant time to set up and require investments in building purchasing functions and capacities. Some national health insurance schemes have not achieved high population coverage, mostly providing coverage for formal-sector employees, who are easily organized under these schemes.^45,68,69^ Employment in many African economies remains largely informal, and the schemes struggle to cover informal-sector workers, who are not as easily organized to collect contributions.^70^ There may be more benefit to countries in building on existing financing arrangements, especially government budget financing, before undertaking major new institutional reforms, which could further fragment health financing arrangements and erode equity. Countries may do better to invest in the improvement of the purchasing functions within existing schemes than create new structures that may not work well in their contexts. Purchasing within government budget financing can be improved within PFM rules, by streamlining spending arrangements and providing more flexibility to providers for planning and financial decisions while harmonizing and strengthening accountability mechanisms.^48^

### Channel Funds to Frontline Providers and Increase Their Autonomy to Respond to Incentives

Although PFM rules are important instruments to guide budget planning, execution, and accountability for public resources,^48^ PFM rules can limit the ability of frontline health care providers to receive funds and use them flexibly to respond to payment incentives. Adapting PFM rules to allow funds to directly reach frontline providers and increase their autonomy to manage those funds can increase the effectiveness of health purchasing levers and improve budget execution.^71^ Some countries, such as Burkina Faso and Tanzania, are making improvements within their PFM rules to overcome these obstacles.

In Tanzania and Uganda, the transfer of funds to lower-level and PHC providers through DHFF and PHC grants, respectively, have increased resource allocation to these levels and away from curative hospital-based services. In both countries, clear guidelines have been provided for planning, budgeting, budget execution, and accountability of these funds by the facilities, which are best placed to identify community needs and priorities. Bottom-up planning to identify priorities and determine allocation of resources to respond to these priorities makes the health facilities more responsive to community needs.

### Build on Existing Information Systems to Generate Evidence for Purchasing Decisions

Many countries have aspirations to develop high-tech ICT solutions to generate evidence for decision making. These systems are often expensive and beyond their current resource availability. In these cases, purchasers can start with existing systems, aim to make them interoperable, and identify a manageable number of indicators (even if they are collected manually or in simple spreadsheets) that provide a broad view of provider performance and the health system. Over time, purchasers can build on existing systems as they create a culture of data use.

Achieving more strategic purchasing that brings results for UHC is a journey. Countries make gradual progress, overcoming challenges with incremental changes that build up to health system improvements. However, there is a need for an overarching strategy with a clear roadmap for these incremental changes. Mapping the purchasing arrangements and functions and tracking progress over time can provide useful information and a policy dialogue tool to prioritize actions and investments and continue to make progress.

## Limitations of the Study

The framework that was applied for this study has limitations in that it focuses primarily on the purchaser and its mandate and capacity to carry out the purchasing functions. The perspectives of the provider and communities are limited because the framework captures only the provider responses to purchaser incentives, factors that limit provider responses to financial incentives, and community participation in benefits specification. Application of the framework relies heavily on document review, and what is captured in policy documents may vary from what is happening in practice. Anyone applying the framework should validate the information in legislation, decrees, government strategies, implementation guidelines, policy reports, and briefs with actual practice at the provider and community levels. Finally, while the framework provides a broad and detailed view of the purchasing arrangements in a country, the result is only a cross-sectional snapshot, and it requires updating over time to reflect progress and changes in purchasing arrangements and associated results. SPARC will continue to iterate on the framework to ensure that it remains relevant in capturing purchasing arrangements regardless of the configuration of the health financing systems.

## Conclusions

The Strategic Health Purchasing Progress Tracking Framework takes the focus off individual schemes and facilitates more practical discussions between technical experts and policy makers to identify priority areas for strengthening strategic purchasing in the system as a whole. This analysis exposed how the fragmentation in financing arrangements severely limits the ability of new purchasing approaches, no matter how sophisticated, to significantly affect service delivery outcomes and progress toward other UHC objectives. While fragmentation and the persistence of high out-of-pocket payments continue to limit the power of strategic purchasing in sub-Saharan Africa, progress is possible. More can be done to improve purchasing within existing budget systems and to learn from and implement new purchasing approaches adopted by PBF and other schemes. The study shows that countries do not have to wait until major consolidation of health financing arrangements becomes politically feasible to take steps to defragment purchasing arrangements and make more effective use of limited government funding for UHC.

## Figures and Tables

**Table 1:** Country health financing indicators.

	Benin	Burkina Faso	Cameroon	Ghana	Kenya	Nigeria	Rwanda	Tanzania	Uganda
Population (millions), 2019*	11.8	20.3	25.9	30.4	52.6	200.9	12.6	58	44.3
GDP per capita (current USD), 2019*	$1,219	$787	$1,508	$2,202	$1,817	$2,230	$820	$1,122	$794
Poverty headcount at $1.90 USD per day (% of population), 2015**	50%	44%	26%	13%	37%	39.1%	57%	49%	42%
Life expectancy at birth (years), 2019**	61	61	59	64	66	54	69	65	63
Current health expenditure (CHE) per capita (current USD), 2018*	$31	$40	$54	$78	$88	$84	$58	$37	$43
Domestic government expenditure as % of CHE, 2018*	20%	43%	6%	37%	42%	15%	31%	43%	16%
External funding as % of CHE, 2018*	30%	15%	9%	12%	16%	8%	31%	32%	43%
Out-of-pocket expenditure as % of CHE, 2018*	45%	36%	75%	37%	24%	77%	11%	24%	38%
Incidence of catastrophic spending (at 25% of household spending)***	5.4% (2012)	0.4% (2011)	3% (2014)	0.1% (2012)	1.5% (2015)	4.1% (2012)	0.1% (2016)	1.2% (2011)	3.8% (2016)
Service coverage index, 2017****	39.6%	39.7%	45.9%	47.4%	55.1%	42.1%	56.9%	43.2%	45.4%

* Global Health Expenditure Database (https://apps.who.int/nha/database) and Rwanda data from the *Rwanda Health Resource Tracking Tool Report FY 2015/16 and 2016/17*.

** World Bank Databank (https://databank.worldbank.org/home.aspx).

*** Global monitoring report on financial protection in health 2019. Geneva (Switzerland): World Health Organization and International Bank for Reconstruction and Development/The World Bank; 2020. [accessed September 29, 2021]. https://www.who.int/data/monitoring-universal-health-coverage.

**** Primary health care on the road to universal health coverage: 2019 monitoring report. Conference edition. Geneva (Switzerland): World Health Organization; 2019. [accessed September 29, 2021]. https://www.who.int/healthinfo/universal_health_coverage/report/uhc_report_2019.pdf.

## Data Availability

The authors confirm that the data supporting the findings of this study are available within the article and/or its supplementary materials.
